# Asymmetric sharing of pollinator fig wasps between two sympatric dioecious fig trees: a reflection of supply and demand or differences in the size of their figs?

**DOI:** 10.1186/s40529-022-00338-9

**Published:** 2022-03-22

**Authors:** Hui Yu, Zhiwei Zhang, Lu Liu, Yufen Cheng, Xiaoxia Deng, Simon T. Segar, Stephen G. Compton

**Affiliations:** 1 School of Life Sciences, Qufu Normal University, Qufu, 273165 Shandong China; 2 Guangdong Provincial Key Laboratory of Applied Botany and Key Laboratory of Plant Resources Conservation and Sustainable Utilization, South China Botanical Garden, The Chinese Academy of Sciences, Guangzhou, 510650 China; 3 Department of Crop and Environment Sciences, Harper Adams University, Newport, TF10 8NB UK; 4 School of Biology, University of Leeds, Leeds, LS2 9JT UK; 5 South China Botanical Garden, The Chinese Academy of Sciences, Xingke Road 723, Tianhe district, Guangzhou, 510650 China

**Keywords:** Host specificity, Hybrid, Mutualism, Pollinator sharing, Size-matching

## Abstract

**Background:**

Host specificity among pollinator fig wasps (Agaonidae) depends on host plant specific volatile cues, but fig wasps must also pass through a narrow physical barrier (the ostiole) if they are to pollinate and oviposit. Across South East Asia the dioecious shrub *Ficus hirta* is associated with at least ten pollinator species allied to *Valisia javana*. *Ficus triloba* has a single recorded pollinator*, Valisia esquirolianae*. Receptive figs of *F. hirta* are usually much smaller than those of *F. triloba*, but at a mainland site where *F. hirta* has atypically large figs we identified both *V. esquirolianae* and *V. javana* from both *Ficus* species using COI and ITS2 sequencing. To investigate whether this host overlap was exceptional we reared fig wasps from the two trees elsewhere and recorded features that may facilitate host transfer between them, including attractant volatiles, reproductive phenology and the sizes of their figs and fig wasps.

**Results:**

The two *Ficus* species were found to support both *Valisia* species at several of the sites, suggesting that the differences we detected in volatile profiles, ostiole sizes and pollinator head sizes are not strict barriers to host sharing. *Valisia javana* colonised *F. triloba* more frequently than *V. esquirolianae* colonised *F. hirta*.

**Conclusions:**

This asymmetric sharing of pollinators may reflect the relative abundance of the two species of fig wasps and differences in host reproductive phenology. Asynchronous flowering of individual *F. hirta* may favor local retention of pollinators, in contrast to the tree-wide synchrony of *F. triloba* figs, which can generate local shortages of *V. esquirolianae.* If the pollinator sharing by male figs of *F. triloba* and *F. hirta* also occurs in female figs then this could result in gene flow between them.

**Supplementary Information:**

The online version contains supplementary material available at 10.1186/s40529-022-00338-9.

## Background

*Ficus* (Moraceae) is one of the most species-rich genera of woody plants in tropical and subtropical regions of the world (Harrison 2005), with more than 800 described species. *Ficus* shares a mutually beneficial symbiotic relationship with pollinating fig wasps (Agaonidae) that has remained structurally unchanged for tens of millions of years (Compton et al. 2010). As a result, fig trees and their pollinators have been a model system for the study the co-evolution and co-speciation between plants and insects (Janzen 1969; Weiblen 2002; Rønsted et al. 2005; Cruard et al. 2012). It was long believed that each species of *Ficus* is pollinated by a single species of fig wasp associated with a single host species, and that it was this specific obligate fig wasp pollination that has promoted both reproductive isolation and speciation in the genus (Ehrlich and Raven 1964; Segar et al. 2019). It is now becoming apparent that this apparent extreme specificity was in part at least a consequence of low sampling intensity in what is a largely tropical mutualism, which often resulted in records that were based on samples of a single species of pollinator from one or a small number of locations (Yu et al. 2021). Widely distributed species of fig trees are increasingly becoming known to support two or more species of pollinators. The insects rarely seem to have sympatric distributions, so in any single location only a single pollinator species tends to be present, except in contact zones (Sun et al. 2011; Chen et al. 2012; Yu et al. 2019). More rarely, examples are being found of pollinators that are not host-plant specific and routinely develop in the figs of two or more host *Ficus* (Lopez-Vaamonde et al. 2002; Cornille et al. 2012; Wei et al. 2014). Host specificity nonetheless remains the norm, and studies of exceptions to a 1:1 relationship provide an opportunity to examine how this specificity is achieved.

Fig wasp host specificity is linked to the unique structure of the *Ficus* inflorescence (the fig or syconium). Figs are formed like a hollow ball, lined on the inside by many tiny flowers, where protogyny prevents geitonogamy (self-pollination resulting from pollen movements between flowers in the inflorescence). The larvae of the fig wasps develop in the galled ovules of female flowers and pollen-carrying adult female pollinators disperse away from their natal figs in order to find and enter receptive figs where they can lay their eggs (Galil and Eisikowitch 1968). Female fig wasps are attracted to the figs by what are typically species specific- and developmental-stage specific blends of volatile compounds released from receptive figs when they are ready for pollination (van Noort et al. 1989; Grison-Pigé et al. 2002; Raguso 2008; Soler et al. 2011; Gu et al. 2012; Wang et al. 2021). Entry is achieved via a small hole called the ostiole which opens for a brief period during receptivity. Successful entry through the ostiole depends on foundresses having an appropriate head shape and body size (van Noort and Compton 1996; Cook and Segar 2010; Liu et al. 2013) and for successful oviposition the female must also have an ovipositor that is longer than the styles through which they reach the ovules where they lay their eggs (Nefdt and Compton 1996; Weiblen 2004). Females of some species re-emerge from the first figs they enter, but they can then only attempt entry into other figs on the same plants, because their wings become detached when they first enter the ostiole (Suleman et al. 2013; Mohd Hatta et al. 2021).

Although inter-specific variation in the physical structure of receptive figs and differences in the volatile cues they emit generate the specificity of the relationship (van Noort et al. 1989; Grison-Pigé et al. 2002), they do not entirely prevent the arrival at receptive figs of pollinators that routinely reproduce inside figs of other species (van Noort et al. 2013) and some of these pollinators manage to penetrate through the ostiole and even successfully reproduce (Compton 1990). Such ‘mistakes’ appear infrequent (Cook and Segar 2010) but may be more common in boundary locations or where a tree’s normal pollinators are rare or absent (Ware and Compton 1992; Compton et al. 2009). Fig trees where there are occasional occurrences of atypical pollinators in their figs can be contrasted with those species that are routinely pollinated by two or more fig wasp species. The trees are usually supporting clusters of sister fig wasp species that appear to have diverged after their relationship with a particular species of fig tree was established (Yu et al. 2019). Based on the data currently available, it appears that only a single species of pollinator is present in each part of their range (Bain et al. 2016; Yu et al. 2019), but there are exceptions (Chen et al. 2012; Rodriguez et al. 2017) and sampling intensities remain low, so views on this may change in the future.

Factors that facilitate sharing of pollinators are likely to include having a complementary flowering phenology, have receptive figs that are of a similar size offering similar visual cues (Lomásco and Levey 2020) and with ostioles of a similar width, and figs that release attractant volatile blends that share common components that the fig wasps use as cues (Liu et al. 2015). Such combinations seem particularly likely to occur in the Asian tropics and sub-tropics, where *Ficus* communities are rich in closely-related species (Berg and Corner 2005), many of which share congeneric pollinators. This was confirmed by our survey of fig wasps reared from closely-related *Ficus* species in Guangdong Province, China where several *Ficus* species were found to be hosts of two or more pollinator species (Yu et al. 2021). Further samples of an atypical population of *F. hirta* Vahl.*,* where the trees produce unusually large figs that are similar in size to those of the related *F. triloba* Buch.-Ham. ex Voigt found that both trees could support two species of *Valisia* fig wasps at the site, one of which we had previously recorded as associated with *F. hirta*, while the other is recorded as the pollinator of *F. triloba* in Taiwan (Chen and Chou 1997).

In order to better understand the basis of fig wasp sharing between *F. hirta* and *F. triloba*, we used sequencing to identify fig wasps breeding in their figs at additional sites in southeast China, and combined this with our previous genetic studies on the pollinators of *F. hirta* to address the following questions (1) How closely related are the fig wasps associated with the two trees? (2) Is sharing asymmetrical, with each fig wasp more abundant in figs of one host than the other? (3) Did the unusually large figs of *F. hirta* at one site facilitate host plant exchange with the pollinator of *F. triloba*? And (4) Which other features of their figs may be facilitating the sharing of fig wasps by the two plants?

## Materials and methods

### Focal species

The mutualism between *F. hirta* and its fig wasp pollinators does not follow the classic one pollinator: one *Ficus* pattern. Recent molecular studies detected nine different closely related *Valisia* species breeding in figs of *F. hirta* in different parts of its extensive range (Yu et al. 2019). Most of the species have not been formally described, and there is uncertainty about which of the currently available names should be applied. *Valisia javana javana* was described from *F. hirta* collected in Java (Mayr 1885) and *Valisia javana hilli* (as *Blastophaga javana hilli*) was described from figs of *F. hirta* collected in Hong Kong (Wiebes 1994; Cruaud et al. 2010). Here for convenience, we use the name ‘*V. javana* sp. 1’ for what is one of several species in this aggregate. In addition to the nine pollinator species already recorded from *F. hirta,* we record here a tenth species, *Valisia esquirolianae*, which has previously been reared from figs of *F. triloba* (as *F. esquirolianae*) in Taiwan (Chen and Chou 1997). *V. esquirolianae* differs morphologically from *V. javana* (Wiebes 1994; Chen and Chou 1997).

*Ficus triloba* and *F. hirta* are closely related dioecious fig species that belong to subsection *Eriosycea* of *Ficus* subgenus *Ficus.* Fig wasps develop in the figs on male trees and seeds develop in figs on female trees. The two species co-exist across most of their widespread ranges that extend throughout northern India, South East Asia and Southern China (Berg et al. 2011). They differ in growth form, with *F. hirta* usually a shrub or a small tree not higher than 5 m and *F. triloba* forming a small tree up to 15 m tall (Berg et al. 2011). The figs of both species are produced in the leaf axils and are more variable in shape and larger in *F. triloba*, reaching about 30 mm in diameter, whereas mature figs from most populations of *F. hirta* reach a diameter of 15 mm. However, at the northern edge of its distribution the diameters of *F. hirta* figs can reach 20 mm (Kuaraksa et al. 2012; Yu et al. 2018). Production of figs by *F. hirta* is asynchronous, with figs of different ages routinely present on the same individuals (Kuaraksa et al. 2012). This allows pollinators emerging from its figs on male plants to potentially re-enter figs on the same trees. In contrast, *F. triloba* individuals usually produce discrete crops of similar-aged figs, which forces its pollinators to fly to other trees though there is also some synchrony among trees. There is nonetheless occasional asynchronous fig production within trees, especially by male plants (Kuaraksa et al. 2012). *F. triloba* can produce up to three crops a year (Kuaraksa et al. 2012). *F. hirta* produces figs year-round, but in the south of China fig production peaks during the wet season from April to October (Yu et al. 2006, 2008).

### Source populations

**Fig. 1:**
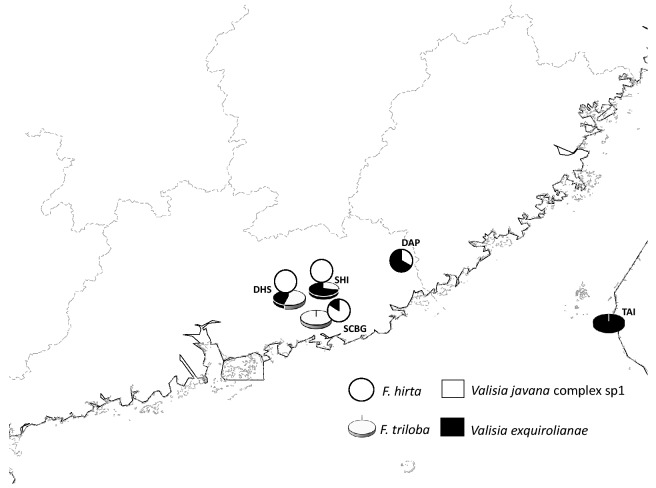
Sample sites and distributions of the pollinator fig wasp species associated with *Ficus hirta* and *F. triloba*

Figs of *F. hirta* in Dapu City (N24°14′19″, E116°49′13″; DAP) of Guangdong province, were collected after it was noted that the figs (of both sexes) on the plants at this site had noticeably larger figs than is typical for this species, closer in size to figs of *F. triloba*. Site DAP has a seasonal climate with a mean annual temperature of around 21.1 °C and January as its coldest month (Liu et al. 2016). *F. triloba* is also present in the area (Fig. 1).

Further samples of *F. hirta*, *F. triloba* and their pollinators were collected from three sites in Guangdong province where the two trees can be found growing close together (Fig. 1). The South China Botanical Garden (N 23° 10′ 46″, E113° 21′ 06″; SCBG) is about 90 km east of Dinghu Mountain (N 23° 09′ 21″, E112° 30′ 39″; DHS). Shimen Mountain (N 23° 38′ 7″, 113°46′ 20″; SHI), is a further 60 km northeast of SCBG. Edaphic and climatic conditions at the three sites are similar with mean annual temperature around 21.5 °C and January the coldest month with a mean of 12.4–13.1 °C (Yu et al. 2006; Han et al. 2019). *Ficus hirta* is an abundant and naturally-occurring species in and around the South China Botanical Garden, whereas we only know of a single (apparently naturally occurring) male individual of *F. triloba* there. Dinghu Mountain has semi-natural vegetation, described as southern subtropical monsoon evergreen broadleaved forest (Han et al. 2019). Both *F. hirta* and *F. triloba* are common there, whereas *F. triloba* is more common than *F. hirta* at Shimen Mountain, an area of mainly secondary forest. The three sites therefore represent a spectrum from *F. hirta* being predominant (South China Botanical Garden) to being the less abundant of the two species (Shimen Mountain).

*F. triloba* was also sampled from Gaoxiong city in Taiwan (N22°53′54″, 120°43′45″; TAI), an island where *F. hirta* is absent (Fig. 1; Tzeng 2004; Bain et al. 2015). Taiwan has a subtropical monsoon climate with a distinct wet season from April to September (Fig. 1).

### Fig wasp collections

Figs were collected from male plants at the developmental stage just before the fig wasps that had developed inside were about to emerge (Galil and Eisikowitch 1968), on dates between 2013 and 2018 (Table 1; Fig. 1). The figs were then placed in net-covered pots and the wasps that emerged were stored in ethanol prior to sequencing. Genetic analyses were based on sequencing of 1–3 fig wasps from each fig and supplemented previous samples from *F. hirta*, from which a total of nine species of *Valisia* pollinators have been recorded across China and South East Asia (Yu et al. 2019). Published phylogenies have shown that the genera *Ceratosolen* and *Kradibia* constitute an outgroup relative to all other pollinating fig wasps (Cruaud et al. 2010). We included representatives of these two genera (two species of *Ceratosolen* and 11 species of *Kradibia*) in our phylogenetic analysis of the *Valisia* pollinators (see Yu et al. 2019).

**Table 1:** The presence of adult offspring of *Valisia javana* complex sp.1 and *Valisia esquirolianae* in male figs of *Ficus hirta* and *F. triloba* collected from Guangdong province, China and Taiwan

Host species	Sites	N trees/figs/ Fig wasps	*V. javana* complex sp.1	*V. esquirolianae*	% *V. javana* complex sp.1
*F. hirta*	South China BG	14/15/19	16	3	84.2
	Dinghu Mountain	8/10/14	14	0	100.0
	Shimen Mountain	2/3/4	4	0	100.0
	Dapu	7/15/15	5	10	33.3
	Total	31/43/52	39	13	75.0
*F. triloba*	South China BG	1/2/ 2	2	0	100
	Dinghu Mountain	12/18/26	14	12	53.8
	Shimen Mountain	8/15/21	6	15	28.6
	Total	21/35/49	22	27	44.9
	Taiwan	1/3/6	0	6	0

Identification was based on COI and ITS2 sequences. Sample sizes indicate how many fig wasps were sequenced and how many figs and trees they were collected from. No *F. triloba* individuals were found at Dapu and *F. hirta* is absent from Taiwan

### *Ficus* species reproductive phenology

The pollinators of dioecious figs appear to disperse less widely than those of monoecious species, reflecting their hosts’ small stature, high local population densities and often asynchronous within-tree fruiting (Harrison and Yamamura 2003; Ahmed et al. 2009; Yang et al. 2015). Any movements of pollinators between different dioecious *Ficus* species are therefore likely to require local overlap in the times when fig wasps are being released from one species and there are receptive figs of a second species available nearby. Weekly phenological censuses of male and female *F. hirta* were conducted at South China Botanical Garden in 2002 and 2003 (Yu et al. 2006) and casual recording suggests that the fruiting there is typical of the species throughout its range. For comparison, we recorded the reproductive phenology of nine male trees and eight female trees of *F. triloba* growing at Dinghu Mountain from August 2017 to July 2018. Recording methods were slightly different because *Ficus triloba* has far larger crops than *F. hirta*. Crop initiation was recorded when at least five figs had appeared and the crop was considered to have ended when no figs remained on the tree. Counts were made of the number of receptive figs and figs from which newly adult fig wasps were emerging.

### Morphological comparisons of pollinator species and their host figs

**Fig. 2:**
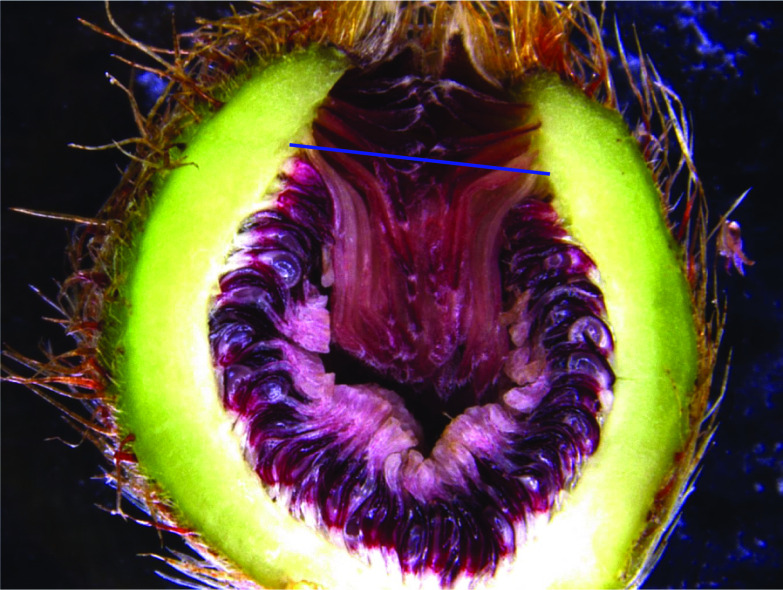
The syconia of *F. hirta* showing the measure of the ostiole diameter. The length of the blue line represent the ostiole diameter (mm)

The diameters of whole receptive male figs of *F. hirta* from the South China Botanical Garden and Dapu City and *F. triloba* from Dinghu Mountain were measured across their equator (at right angles to the ostiole) to the nearest 0.1 mm using vernier calipers. For each species, at least five figs from each individual and five or more individuals were measured. An indication of the size of the ostioles through which the insects needed to crawl was obtained by halving receptive male figs from the centre of the ostiole to the centre of attachment of the basal peduncle. The distance between the descending bracts at their widest part (Fig. 2) was measured to the nearest 0.1 mm using photographs taken using a Leica E24W camera. Female fig wasps entering the figs pass through the centre of the descending bracts and this measurement provides an indication of the extent to which the bracts could be pushed aside. The calibrated digital images were then recorded using Imagej (version 1.48) software. Flowers were removed haphazardly from male figs to measure their styles, with the lengths of the styles measured from where the style joins the ovary to the tip of the stigma (Nefdt and Compton 1996; Yu and Compton 2012). Photographs and Imagej software were used for this. For each species, we measured the female flowers and pollinators obtained from at least five figs from each of five or more individuals.

Head shape and size in female fig wasps is likely to be under selection pressure generated by the size of the ostioles through which they need to crawl if they are to oviposit (van Noort and Compton 1996; Liu et al. 2013). We measured the size of the heads of female fig wasps reared from *F. hirta* and *F. triloba* figs that had been stored in 70% ethanol. The maximum head lengths and widths (across the compound eyes) were measured under a Leica E24W microscope and again using Imagej software (version 1.48). After successful passage through the ostiole, female fig wasps can only lay eggs if their ovipositors are of sufficient length to reach the ovules where the eggs are placed (Nefdt and Compton 1996). The lengths of ovipositors (first and second valvulae) of fig wasps reared from the two host species were measured using the same software after they had been dissected out and placed under a cover slip.

### Volatiles emitted by receptive male figs

Floral volatiles released by receptive figs were collected in situ from eight male individuals of *F. hirta* and five male individuals of *F. triloba* growing in Dinghu Mountain using the chromatoprobe head-space technique, following methods developed for other fig tree species (Proffit and Johnson 2009; Soler et al. 2012). Samples were analyzed with gas chromatography–mass spectrometry (GC–MS) using a GC–MS QP2010Plus (Shimadzu Instrument Co., Ltd; Japan) following the procedures of Soler et al. (2012). Compounds were mainly identified by matching of mass spectra with the FFNSC1.3 and NIST14S libraries, and by comparison of Kovats retention indices with that reported in the NIST chemistry Web Book (http://webbook.nist.gov) and published data. The peak area of each compound was used for quantification of the relative quantities of each component and was calculated using the normalization method (Soler et al. 2012).

### Pollinator identification

Total genomic DNA was extracted from whole bodies of the fig wasps using the EasyPure Genomic DNA Extraction Kit (TransGen, Beijing, China). A 587–689 bp fragment of the mtCOI gene for each pollinating species was then sequenced following the protocol used in previous studies (Tian et al. 2015). The reaction was optimized and programmed on a MJ Thermal Cycler (PTC 200) with one cycle of denaturation at 94 °C for 5 min, 35 cycles of 30 s denaturation at 94 °C, 30 s at a 55 °C annealing temperature, and 30 s extension at 72 °C, followed by 8 min extension at 72 °C. A 689 bp fragment of the ITS2 gene was amplified in 50 individuals of *V. javana* complex sp.1 and 16 individuals of *V. esquriolianae* collected in SCBG, DHS, SHI and DAP using the universal primer pair (ITSR: 5′‐CGCCTGATCTGAGGTCGTGA‐3′, ITSF: 5′ATTCCCGCACCACG CCTGGCTGA‐3′; Lopez-Vaamonde et al. 2001) and the same PCR amplification reaction as for the COI gene. The amplified PCR products were purified using QIAquick spin columns (Qiagen) and were sequenced in an ABI 3730xl capillary sequencer using BigDye Terminator V 3.1 chemistry (Applied Biosystems). All unique haplotype sequences were deposited in GenBank (accession numbers: MT145612-MT145639 for COI sequences and MT218428 for *V. esquirolianae* ITS2 sequence). There was only one ITS2 haplotype recorded for each species. The ITS2 haplotype recorded here under ‘*V. javana’* was the same as that of *V. javana* complex sp.1 published in Yu et al. (2019) in Genbank with accession number MF467418.

We did not detect any indication of pseudo-genes, such as multiple peaks in chromatograms, stop codons or frame shift mutations (Song et al. 2008). Sequences were aligned using MUSCLE (Edgar 2004) implemented in MEGA 6.0 (Tamura et al. 2013) with manual corrections. DnaSP was used to count the number of haplotypes (Librado and Rozas 2009). Neighbour-joining trees were constructed using MEGA 6.06 (Tamura et al. 2013) for both COI and ITS2, and node supports were assessed based on 2000 bootstrap replicates. Finally, Kimura-2-parameter (K2P) distances within and between clades for both COI and ITS2 haplotypes were calculated.

### Statistical analysis

Between-site and -species differences in fig diameter, ostiole diameter, style length, and their pollinating wasp ovipositor lengths, head width and head length were examined (after testing for equality of variance) with independant sample t-tests in SPSS 21.0.

Statistical analyses of volatile data were performed using past 3.0. In order to compare patterns of odour composition between two species we performed a nonmetric multidimensional scaling (NMDS). For the comparison of volatile profiles between two species we used the relative proportions of all the compounds emitted by them. A data matrix of pairwise comparisons among samples was then calculated using the Bray Curtis distance index. NMDS was used to find the best low dimensional representation of the distance matrix. The null hypothesis of no difference in patterns of odour composition between species was tested with a permutational multivariate analysis of variance (PERMANOVA) with 9999 permutations.

## Results

### Reproductive phenology

**Fig. 3:**
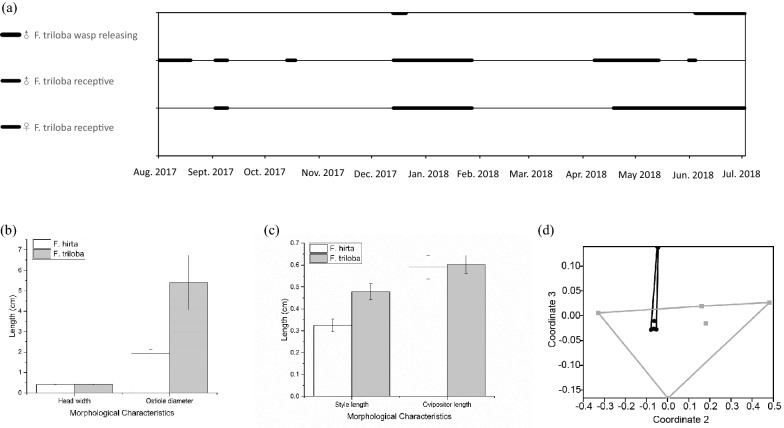
**a** The reproductive phenology of nine male and eight female *F. triloba* at Dinghu Mountain. Each thick bar shows a particular developmental stage present on at least one of the plants. Emerging (D phase sensu Galil and Eisikowitch 1968) phase male figs release pollinator fig wasps, which enter receptive (B) phase male and female figs where they pollinate and attempt oviposition. In contrast to *F. triloba*, fig production on each male and female *F. hirta* individual is asynchronous and small numbers of receptive figs and male figs that are releasing pollinators were present almost throughout the year at this site. **b** Comparison of ostiole diameter in male receptive syconia between *F. hirta* and *F. triloba*, and head width between their specific pollinating wasps. **c** Comparison of style length in male receptive syconia between *F. hirta* and *F. triloba*, and ovipositor length between their specific pollinating wasps. **d** Non-metric multidimensional scaling of receptive floral volatiles of *F. hirta* (black circles) and *F. triloba* (grey squares) based on Bray–Curtis dissimilarity index (stress = 0.128).

Small numbers of figs of varying developmental stages were present throughout the year on the figs of *F. hirta* at Dinghu Mountain. The nine male *F. triloba* trees at Dinghu Mountain all had figs releasing adult fig wasp offspring during short periods in June–July and again in January (Fig. 3a). In contrast, receptive figs on trees of both sexes of *F. triloba* were present for extended periods, especially in the wet season from April to September (Fig. 3a). The extended periods when un-entered receptive figs were present suggests that only limited numbers of pollinators were finding and entering the figs of *F. triloba*. Consequently, receptive male and female figs of *F. triloba* were often available when both *V. esquirolianae* and *V. javana* sp. 1 were emerging from host figs of both *F. triloba* and *F. hirta*.

### Morphology of receptive figs and fig wasp foundresses

The receptive phase male figs of *F. hirta* in South China Botanic Garden were much smaller in diameter than those of *F. triloba* (Table 2, T = 23.35, P < 0.001) and as expected, the diameter of receptive male figs of *F. hirta* from Dapu City were larger than those from South China Botanic Garden (Table 2, T = 2.96, P = 0.004). The median diameter of receptive male *F. hirta* figs from Dapu City was nonetheless still smaller than the figs of *F. triloba* from the same location (Table 2, T = 9.83, P < 0.001), although there was a small overlap in their diameters not seen elsewhere (Table 2). Measurements of the ostiole bract separation showed a similar pattern as the overall diameters of the figs (Table 2; Fig. 3b), with figs of *F. triloba* having significantly longer ostiole widths than those of *F. hirta* at both South China Botanical Garden and Dapu City, despite the ostioles of *F. hirta* figs being wider at Dapu City than the Garden (T tests, all P < 0.05). This might have made it easier for female *V. esquirolianae* that had developed in *F. triloba* figs to enter the figs of *F. hirta* at Dapu City, if they were significantly larger than those of *V. javana* sp. 1. However, although *V. esquirolianae* females were found to have a slightly wider median head width than the *V. javana* sp. 1 reared from *F. hirta* at the same site there was almost complete overlap in head sizes between the two species (Table 3; Fig. 3b).

**Table 2:** Functional morphological features of receptive phase male figs of *Ficus hirta* at sites SCBG and DAP and *Ficus triloba* at site DHS

*Ficus*	Median fig diameter Range (mm) (n)	Median ostiole diameter Range (mm) (n)	Median style length Range (mm) (n)
*F. hirta* (SCBG)	13.03 10.25–15.96 (46)	1.90 1.59–2.42 (49)	0.319 0.272–0.386 (49)
*F. triloba*	26.0 18.85–30.31 (45)	5.09 3.43–5.64 (45)	0.477 0.369–0.549 (42)
*F. hirta* (DAP)	14.65 11.07–19.86 (10)	2.42 1.58–4.43 (10)	–

Style length measurements were made from female flowers in the male figs. (n) indicates the number of measurements

**Table 3:** Measurements of adult female fig wasps reared from figs of *Ficus hirta* and *Ficus triloba*

*Valisia*	Host	Median head width (mm) Range (n)	Median head length (mm) Range (n)	Median ovipositor length (mm) Range (n)
*V. javana* sp.1	*F. hirta*	0.41 0.37–0.47 (34)	0.39 0.35–0.44 (37)	0.59 0.46–0.78 (37)
*V. esquirolianae*	*F. triloba*	0.42 0.39–0.47 (30)	0.40 0.29–0.46 (33)	0.59 0.55–0.70 (28)

(n) = the number of individuals measured. *V. esquirolianae* had significantly wider heads than *V. javana* sp. 1 (T = 2.76, p = 0.008), but the maximum head widths recorded were similar in the two species. There was no significant difference in their head widths (T = 0.88, P = 0.382) nor the lengths of their ovipositors (T = 1.09, P = P = 0.281)

The median style lengths in male figs of *F. hirta* were significantly shorter than those of *F. triloba*, but with considerable overlap between the two *Ficus* species (Table 2, T = 22.11, P < 0.001; Fig. 3c). This difference seems unlikely to differentially impact their associated fig wasps, because the ovipositor lengths of the two species showed complete overlap (Table 3; Fig. 3c).

### Volatiles emitted by receptive male figs

The volatile profiles of *F. hirta* and *F. triloba* both contained more than 50 compounds, with sesquiterpenes predominant in both species (Table 4 and Additional file 1: Table S1). Fully 48 of the compounds were shared by the two *Ficus* species and although several of the most abundant volatiles in the profile from *F. hirta* were relatively less abundant among the volatiles detected from *F. triloba*, and vice versa, there were three common abundant compounds shared by the two species (Table 4). (E)-Caryophyllene was the most abundant compound released by *F. hirta* and was the second by *F. triloba*. Alpha copaene was the second most abundant compound released by *F. hirta* and was the most by *F. triloba* and has been detected previously among the volatiles released by figs of *F. fulva* Reinw. ex Blume (Grison et al. 1999) which is in the same section as *F. hirta* and *F. triloba*. We do not know which of the 50 + compounds are attractive (or repellant) to the two pollinators of *F. hirta* and *F. triloba*, but it is clear that with their almost total overlap in the volatiles they were releasing, there is considerable scope for cross attraction of the two fig wasp species.

**Table 4:** The ten most abundant compounds identified in the volatile profiles of eight receptive male figs of *Ficus hirta* and five receptive male figs of *Ficus triloba* growing at Dinghu mountain

Compound	*F. hirta*	*F. triloba*	Compound	*F. triloba*	*F. hirta*
	Abundance ranking (frequency)	Abundance ranking (frequency)		Abundance ranking (frequency)	Abundance ranking (frequency)
(E)-Caryophyllene	1 (1–2; 8/8)	2 (1–2; 5/5)	α-Copaene	1 (1–3; n = 5/5)	2 (2–4; n = 8/8)
α-Copaene	2 (2–4; n = 8/8)	1 (1–3; 5/5)	(E)-Caryophyllene	2 (1–2; n = 5/5)	1 (1–2; n = 8/8)
α-Funebrene	3 (2–34; n = 7/8)	52 (49; 1/1)	Germacrene D	3 (2–11; n = 4/5)	15 (5–30; n = 7/8)
α-Humulene	4 (1–4; n = 8/8)	12 (2–11; 2/5)	Cyclosativene	4 (2–35; n = 5/5)	6 (4–31; n = 8/8)
β-Elemene	5 (1–14; n = 8/8)	16 (4–23; 4/5)	cis-4(14),5-diene-Muurola	5 (2–30; n = 3/5)	14 (5–30; n = 8/8)
Cyclosativene	6 (4–31; n = 8/8)	4 (2–35; n = 5/5)	unknown 1428	6 (2–13; n = 3/5)	Absent (0/8)
δ-Cadinene	7 (5–38; n = 8/8)	15 (27–40; n = 3/5)	Bicyclogermacrene	7 (2–35; n = 4/5)	29 (14–30; n = 5/8)
α-Guaiene	8 (5–14; n = 7/8)	52 (51; n = 1/5)	2-Propenoic acid,2-methyl-, hexyl ester	8 (2; n = 1/5)	Absent (0/8)
α-Muurolene	9 (4–32; n = 8/8)–	28 (14–20; n = 2/5)	unknown 1446	9 (2; n = 2/5)	Absent (0/8)
Calarene	10 (5–21; n = 8/8)	34 (16–44; n = 2/5)	Limonene D	10 (2–44; n = 4/5)	Absent (0/8)

Each fig was from a different parent tree

The volatile profiles of *F. hirta* and *F. triloba* display overlap using NMDS but there was significant difference between them (PERMANOVA, F = 5.944; P = 0.0009; Fig. 3d).

### The pollinators of *F. hirta* and *F. triloba*

**Fig. 4:**
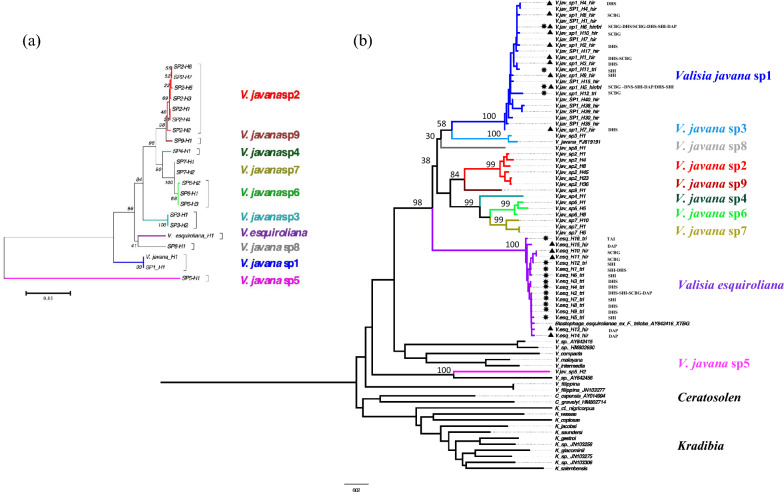
**a** ITS2 neighbour joining tree of the *Valisia* fig wasp pollinators associated with *Ficus hirta* and *Ficus triloba*. **b** COI neighbour-joining tree of the pollinators associated, with sequences of *Ceratosolen* and *Kradibia* species as outgroups. Node support rates are shown. Haplotypes (*V. jav*_sp1_*H1*-*H12* for *Valisia javana* complex sp 1 and *V. esq*_ *H1*-*H16* for *V. esquirolianae*) are also listed together with their host figs (*hir* = *F. hirta* (black triangles), *tri* = *F. triloba* (black stars), *hirt/tri* = both *F. hirta* and *F. triloba*). The collecting sites of their hosts listed by haplotypes. For haplotypes of *Valisia javana* complex sp 1, those sequenced in this study is marked as *sp 1*, the others extracted from Yu et al. (2019) are marked as *SP1*

Adult offspring of the same two species of fig wasps were reared from male figs of both *F. triloba* and *F. hirta* in mainland China and there were even examples of the same fig wasp haplotypes being recorded from both host plants. One of the species was also reared from *F. triloba* in Taiwan and was identified as *V. esquirolianae* (Chen and Chou 1997), where *F. hirta* is absent. These individuals combined with those in the mainland from both hosts formed a single clade that nested with the *V. esquirolianae* reared previously from *F. triloba* in Xishuangbanna (Jiang et al. 2006; Figs. 1, 4; Additional file 1: Tables S2, S3). The second pollinator reared from both host species was identified as being the *V. javana* sp.1 recorded by Yu et al. 2019. *Ficus hirta* therefore supports the development of a tenth pollinator species in addition to the nine species in the *V. javana* complex that have been recorded previously (Fig. 4). *V. esquirolianae* is closely related to the members of *V. javana* complex (Fig. 4), but the relationships among *V. javana* sp.1, *V. e*s*quirolianae* and other species in this complex are unclear because of low bootstrap values (Fig. 4).

At sites where both *Ficus* species were sampled the two species of fig wasps were usually associated with male figs of both host plants, though their relative frequencies varied between sites and hosts (Table 1; Fig. 1). We did not sample *F. triloba* at Dapu City, but approximately equal frequencies of the two fig wasps were present in figs of *F. hirta* there. At South China Botanical Garden, where *F. triloba* is very rare, *F. hirta* figs mainly contained adult offspring of *V. javana* sp.1 and only a single fig of this species contained *V. esquirolianae*. In contrast the small number of sampled *F. triloba* figs sampled contained only *V. javana* sp.1. At Dinghu Mountain, where both plant species are common, *V. javana* was the only species recorded from *F. hirta*, whereas approximately equal frequencies of this species and *V. esquirolianae* were recorded from figs of *F. triloba*. At Shimen Mountain *V. esquirolianae* was the more frequent occupier of *F. triloba* figs (71.4% of the total), but our small sample of *F. hirta* figs only recorded *V. javana* offspring. The relative abundance of the two *Valisia* species therefore varied considerably between sites in mainland China, but across mainland sites as a whole 74.4% of the 43 male figs of *F. hirta* had supported the development of *V. javana*, whereas only 42.3% of the 35 *F. triloba* figs had supported this species. *V. esquirolianae* was therefore less frequent overall and Dapu was the only site where a majority of *F. hirta* figs had supported this species. In Taiwan, where *F. hirta* is absent, no *V. javana* offspring were recorded in the six *F. triloba* figs we sampled.

## Discussion

We reared the same two fig wasp species from male figs of *F. hirta* and *F. triloba* at most of the Guangdong province sites we investigated. Our hypothesis that figs of *F. hirta* at Dapu City were supporting development of *V. esquirolianae* because its figs were larger there than elsewhere can therefore be rejected, though *F. hirta* at this site did support more *V. esquirolianae* than elsewhere. Monitoring of fig wasp entry success into figs of different sizes will be required to determine whether penetration by either *Valisia* species is influenced by their head widths relative to ostiole sizes (Liu et al. 2013).

The shared use of the figs of the two *Ficus* species is likely to have been facilitated by individuals of the two species sometimes growing in close proximity, their complementary fruiting phenologies and the rather similar volatile blends released by their receptive figs, possibly together with the overlap in sizes of the two fig wasp species. Studies involving electroantennogram and behavioral responses of the two fig wasps to known volatiles are required before an assessment can be made of the extent to which the two *Ficus* species share functionally significant volatiles. Despite *F. triloba* producing larger figs, the style lengths of its female flowers also overlapped with those inside the smaller figs of *F. hirta*, so after they enter male figs the two *Valisia* species are likely to find oviposition equally easy.

Our results suggest that transfer of *V. javana* females into *F. triloba* figs was more frequent than the entry of *V. esquirolianae* into figs of *F. hirta.* The overlap in size of the two pollinators suggests that the marginally larger heads of *V. esquirolianae* are unlikely to be biologically significant. Alternatively, the asymmetry in host transfer could be a simple reflection of the relative abundance of the two fig wasps, rather than indicating differences in their willingness to enter figs of an unusual host. Differences in the trees’ flowering patterns may also be significant. The flowering phenology of *F. hirta* is typical of many dioecious shrubby *Ficus* species, with asynchronous development on individual trees. Figs at different developmental stages are therefore present in close proximity, with fig wasps being released nearly all year round and providing opportunities for emerging fig wasps to enter receptive figs on the same male trees (Yu et al. 2006). This is not the case with *F. triloba,* where more synchronous crops will require movements of pollinators between trees. Furthermore, at the single site where we monitored the phenology of this species we found receptive figs were present for extended periods. This suggests a local shortage of *V. esquirolianae,* a situation that is likely to make entry of atypical pollinator species more likely (Compton 1990).

The distributions of *F. hirta* and *F. triloba* overlap across much of their extensive ranges. *Ficus hirta* is absent from the island of Taiwan, where *V. esquirolianae* is the only pollinator recorded for *F. triloba* so far. Whether the distribution of *V. esquirolianae* extends to the south of China and southeast Asia are unknown, but extensive sampling of 530 fig wasps from *F. hirta* in at least 34 locations for did not detect any *V. esquirolianae* (Yu et al. 2019), but did record a further eight *Valisia* species. It seems possible that one or more of these species is associated *F. triloba* as well as *F. hirta*, and might replace *V. esquirolianae* in the southern part of its range.

In plants pollinated by insects, their host specificity and behavior can have a major role in determining the extent to which pollen is transferred between individuals and consequently the extent of gene flow between them (Peter and Johnson 2014). We only confirmed the ability of the two fig wasp species to successfully reproduce in the male figs of the two *Ficus* species, and that sharing of the two fig wasps is widespread. It is nonetheless highly likely that female fig wasps that had developed in either host were also able to enter female figs of both *F. hirta* and *F. triloba* irrespective of the identity of their natal figs. Whether heterospecific pollen carried into the figs could result in the production of viable seed is unknown, but post-pollination barriers to hybrid production are weak in *Ficus* (Ghana et al. 2015; Wang et al. 2016)*.* The pollinator sharing of *F. triloba* with *F. hirta* therefore has the potential to result in gene flow between them (Arnold 1997; Coyne and Orr 2004).

## Supplementary Information

**Additional file 1: Table S1.** Tentative results of relative abundances (RA%, Mean ± SD) and occurrences (O) of the compounds in the volatile profiles of receptive syconia collected from *Ficus hirta* and *Ficus triloba* in Dinghu Mountain. Zeros indicate that the compound was not recorded. **Table S2.** Pollinating wasp COI cytoplasmic gene sequence differences (Kimura-2-parameter) within (diagonal) and between species (below diagonal). Sequence differences of between *V. esquirolianae* (*V. esq*) and *V. javana* pollinating wasps (sp 1, 2, 3, 4, 5, 6, 7, 8, 9) were high (highlighted in yellow) what imply they are different species. **Table S3.** Pollinating wasp ITS2 nuclear gene sequence differences (Kimura-2-parameter) within (diagonal) and between species (below diagonal). Sequence differences of between *V.esquirolianae* (*V. esq*) and the other *V. javana* pollinating wasps (sp1, 2, 3, 4, 5, 6, 7, 8, 9) were high (highlighted in yellow) what imply they are different species.

## Data Availability

All unique haplotype sequences were deposited in GenBank (accession numbers: MT145612-MT145639 for COI and MT218428 for ITS2).

## Abbreviations

SCBG: South China Botanical Garden
DNA: Deoxyribonucleic acid
mtCOI: Mitochondrial cytochrome oxidase I
COI: Cytochrome oxidase I
K2P: Kimura-2-parameter
