# Supplementary material for: Asymmetric sharing of pollinator fig wasps between two sympatric dioecious fig trees: a reflection of supply and demand or differences in the size of their figs?
Source: Bot Stud. 2022 Mar 22;63:7. doi: 10.1186/s40529-022-00338-9 (PMC8941105; doi:10.1186/s40529-022-00338-9)
Supplement: Supplementary file 1 — Additional file 1: Table S1. Tentative results of relative abundances (RA%, Mean ± SD) and occurrences (O) of the compounds in the volatile profiles of receptive syconia collected from Ficus hirta and Ficus triloba in Dinghu Mountain. Zeros indicate that the compound was not recorded. Table S2. Pollinating wasp COI cytoplasmic gene sequence differences (Kimura-2-parameter) within (diagonal) and between species (below diagonal). Sequence differences of between V. esquirolianae (V. esq) and V. javana pollinating wasps (sp 1, 2, 3, 4, 5, 6, 7, 8, 9) were high (highlighted in yellow) what imply they are different species. Table S3. Pollinating wasp ITS2 nuclear gene sequence differences (Kimura-2-parameter) within (diagonal) and between species (below diagonal). Sequence differences of between V.esquirolianae (V. esq) and the other V. javana pollinating wasps (sp1, 2, 3, 4, 5, 6, 7, 8, 9) were high (highlighted in yellow) what imply they are different species. [file 40529_2022_338_MOESM1_ESM.docx]

Table S1. Tentative results of relative abundances (RA%, Mean ± SD) and occurrences (O) of the compounds in the volatile profiles of receptive syconia collected from *Ficus hirta* and *Ficus triloba* in Dinghu Mountain. Zeros indicate that the compound was not recorded.

| Family | Compounds | Kovats RI | *F. hirta* (n=8) | | *F. triloba* (n=5) | |
| --- | --- | --- | --- | --- | --- | --- |
|  |  |  | RA% | O | RA% | O |
| Fatty acid derivatives | | | | | | |
|  | Hex-(3Z)-enol | 857 | 0 | 0 | 0.02±0.04 | 1 |
|  | Vinyl amyl carbinol | 960 | 0 | 0 | 0.01±0.03 | 1 |
|  | n-Decane | 997 | 0 | 0 | 1.84±4.11 | 1 |
|  | Hex-(3Z)-enyl acetate | 1006 | 0 | 0 | 0.26±0.58 | 1 |
|  | 2-Propenoic acid, 2-methyl-, hexyl ester | 1063 | 0 | 0 | 2.66±5.95 | 1 |
|  | Tridecane | 1297 | 0 | 0 | 0.26±0.36 | 2 |
|  | (3Z)-hexenyl-Tiglate | 1324 | 0 | 0 | 0.01±0.02 | 1 |
|  | Cyclooctacosane | 1550 | 0.01±0.02 | 3 | 0.002±0.004 | 1 |
| Benzenoids | | | | | | |
|  | o-Cymene | 1020 | 0.10±0.13 | 3 | 0.004±0.009 | 1 |
|  | Clorius | 1093 | 0 | 0 | 0.02±0.05 | 1 |
|  | (isocyanomethyl)-Benzene | 1217 | 0 | 0 | 1.84±4.12 | 1 |
|  | Prodox 146 | 1509 | 0 | 0 | 0.02±0.04 | 1 |
| Nitrogen containing compounds | | | | | | |
|  | Indole | 1293 | 0 | 0 | 0.01±0.01 | 1 |
| Monoterpenes | | | | | | |
|  | α-Thujene | 924 | 0.09±0.16 | 2 | 0 | 0 |
|  | α-Pinene | 931 | 0.72±0.46 | 8 | 2.28±4.73 | 2 |
|  | Camphene | 945 | 0.07±0.2 | 1 | 0 | 0 |
|  | Dehydrosabinene | 951 | 0 | 0 | 0.13±0.29 | 1 |
|  | β-Pinene | 973 | 0.27±0.31 | 5 | 0.004±0.01 | 1 |
|  | Sabinene | 978 | 0 | 0 | 1.92±2.87 | 2 |
|  | D-Limonene | 1025 | 0 | 0 | 2.55±4.32 | 4 |
|  | (Z)-β-Ocimene | 1037 | 0 | 0 | 0.01±0.01 | 1 |
|  | (E)-β-Ocimene | 1047 | 0 | 0 | 0.09±0.19 | 1 |
|  | 4-Octanol, 4,5-dimethyl | 1053 | 0 | 0 | 0.02±0.05 | 1 |
|  | γ-Terpinene | 1054 | 0.40±0.78 | 4 | 0.06±0.14 | 1 |
|  | Linalool | 1097 | 0 | 0 | 0.05±0.11 | 1 |
|  | 4,8-dimethyl-(E)-Nona-1,3,7-triene | 1116 | 0 | 0 | 0.01±0.02 | 1 |
|  | 1,3,8-p-Menthatriene | 1130 | 0 | 0 | 0.01±0.02 | 1 |
|  | Camphor | 1141 | 0 | 0 | 0 | 0 |
|  | unknown 1173 | 1173 | 0 | 0 | 0.55±1.23 | 1 |
|  | trans-Linalool oxide | 1184 | 0 | 0 | 0.37±0.82 | 1 |
| Sesquiterpenes | | | | | | |
|  | unknow 1301 | 1301 | 0 | 0 | 0.23±0.29 | 3 |
|  | unknown 1330 | 1310 | 0 | 0 | 0.58±0.53 | 4 |
|  | δ-Elemene | 1330 | 0.48±0.28 | 0 | 0.66±0.42 | 5 |
|  | α-Cubebene | 1339 | 0.72±0.37 | 8 | 1.32±2.94 | 1 |
|  | unknown 1350 | 1350 | 0 | 0 | 0.02±0.04 | 1 |
|  | α-Longipinene | 1354 | 0 | 0 | 1.39±1.18 | 5 |
|  | Cyclosativene | 1357 | 1.87±1.56 | 6 | 2.95±2.78 | 5 |
|  | Ylangene | 1370 | 0.11±0.15 | 6 | 0.11±0.20 | 2 |
|  | Isoledene | 1372 | 0 | 0 | 0.40±0.76 |  |
|  | α-Copaene | 1376 | 9.13±3.26 | 8 | 24.0±21.98 | 5 |
|  | Daucene | 1380 | 1.32±1.8 | 4 | 0.75±1.09 | 2 |
|  | β-Bourbonene | 1385 | 0.33±0.24 | 7 | 0.74±0.80 | 5 |
|  | β-Cubebene | 1390 | 0.03±0.05 | 2 | 0.96±1.27 | 3 |
|  | β-Elemene | 1392 | 3.92±4.84 | 8 | 1.64±1.30 | 4 |
|  | Cedrene | 1394 | 0.23±0.61 | 1 | 0.13±0.25 | 2 |
|  | α-Funebrene | 1399 | 8.45±9.11 | 7 | 0.01±0.02 | 1 |
|  | α-Gurjunene | 1407 | 0 | 0 | 0.20±0.44 | 1 |
|  | Isocaryophyllene | 1408 | 0.04±0.12 | 1 | 0.32±0.50 | 2 |
|  | Longifolene | 1409 | 1.19±0.94 | 8 | 0.20±0.46 | 1 |
|  | β-Maaliene | 1411 | 0.37±0.14 | 8 | 0.04±0.08 | 1 |
|  | α-Cedrene | 1413 | 0.02±0.03 | 3 | 0 | 0 |
|  | α-cis-Bergamotene | 1415 | 0.02±0.06 | 1 | 0 | 0 |
|  | (E)-Caryophyllene | 1421 | 49.25±10.45 | 8 | 20.19±13.96 | 5 |
|  | unknown 1422 | 1422 | 0 | 0 | 0.58±0.80 | 2 |
|  | unknown 1428 | 1428 | 0 | 0 | 2.84±3.25 | 3 |
|  | Calarene | 1433 | 1.34±0.75 | 8 | 0.34±0.70 | 2 |
|  | γ-Elemene | 1434 | 1.24±0.96 | 6 | 0.004±0.01 | 1 |
|  | α-trans-Bergamotene | 1436 | 0.02±0.04 | 1 | 0.03±0.07 | 1 |
|  | α-Guaiene | 1439 | 1.64±0.85 | 7 | 0.004±0.01 | 1 |
|  | Isogermacrene D | 1446 | 0.09±0.11 | 3 | 1.42±1.2 | 5 |
|  | unknown 1448 | 1448 | 0 | 0 | 2.64±3.67 | 2 |
|  | epsylon-Muurolene | 1450 | 0.03±0.07 | 1 | 0.93±2.03 | 2 |
|  | α-Humulene | 1455 | 7.12±2.51 | 8 | 1.94±3.22 | 2 |
|  | (E)-β-Farnesene | 1456 | 0 | 0 | 0.38±0.86 | 1 |
|  | Cadina-1(6),4-diene | 1457 | 0 | 0 | 0.15±0.22 | 2 |
|  | 9-epi-(E)-Caryophyllene | 1462 | 0.27±0.41 | 4 | 1.22±1.80 | 2 |
|  | cis-Muurola-4(14),5-diene | 1464 | 1.13±0.58 | 8 | 2.87±3.86 | 3 |
|  | Cadina-1(6),4-diene | 1467 | 0 | 0 | 0.15±0.22 | 2 |
|  | β-Acoradiene | 1470 | 0.03±0.05 | 4 | 0.01±0.01 | 1 |
|  | γ-Muurolene | 1478 | 0.55±0.25 | 8 | 1.29±0.81 | 5 |
|  | Germacrene D | 1483 | 0.76±0.58 | 7 | 4.11±2.93 | 4 |
|  | (Z)- α-Bisabolene | 1487 | 0 | 0 | 0.18±0.39 | 1 |
|  | β-Selinene | 1488 | 0.30±0.27 | 5 | 0 | 0 |
|  | β-Vatirenene | 1490 | 0.01±0.02 | 1 | 0 | 0 |
|  | γ-Amorphene | 1494 | 0.04±0.04 | 5 | 0.02±0.32 | 3 |
|  | α-Selinene | 1497 | 0.17±0.4 | 2 | 0 | 0 |
|  | Bicyclogermacrene | 1498 | 0.24±0.25 | 5 | 2.83±4.34 | 4 |
|  | α-Muurolene | 1500 | 1.55±1.22 | 8 | 0.61±0.87 | 2 |
|  | α-Bulnesene | 1507 | 0.25±0.23 | 5 | 0 | 0 |
|  | (E,E)-α-Farnesene | 1508 | 0.14±0.3 | 3 | 0.20±0.44 | 1 |
|  | γ-Cadinene | 1515 | 0.62±0.64 | 8 | 0.21±0.26 | 3 |
|  | δ-Cadinene | 1524 | 1.71±1.6 | 8 | 1.67±2.12 | 4 |
|  | trans-Cadina-1,4-diene | 1534 | 0 | 0 | 0.03±0.03 | 3 |
|  | β-Cubebene | 1537 | 0 | 0 | 0.05±0.12 | 1 |
|  | α-Cadinene | 1539 | 0.05±0.05 | 6 | 0.01±0.02 | 1 |
|  | (E, E)-α-Calacorene | 1544 | 0.07±0.11 | 4 | 0.12±0.17 | 2 |
|  | 61847-19-6 | 1548 | 0.02±0.02 | 3 | 0.01±0.03 | 1 |
|  | Germacrene B | 1561 | 0.42±0.78 | 6 | 0.06±0.07 | 3 |
|  | β-Calacorene | 1565 | 0.03±0.05 | 3 | 0.03±0.06 | 1 |
|  | Neryl isovalerate | 1578 | 0.04±0.07 | 3 | 0.03±0.07 | 1 |
|  | Caryophyllene oxide | 1585 | 0.01±0.02 | 3 | 0.01±0.02 | 1 |
|  | β-Elemenone | 1603 | 0.01±0.02 | 1 | 0 | 0 |
|  | Humulene epoxide II | 1611 | 0.01±0.02 | 2 | 0.01±0.02 | 1 |
|  | Cedranone | 1615 | 0.58±0.86 | 3 | 0 | 0 |
|  | Longipinocarvone | 1623 | 0.39±0.77 | 3 | 0 | 0 |

Table S2. Pollinating wasp COI cytoplasmic gene sequence differences (Kimura-2-parameter) within (diagonal) and between species (below diagonal). Sequence differences of between *V. esquirolianae* (*V. esq*) and *V. javana* pollinating wasps (sp 1, 2, 3, 4, 5, 6, 7, 8, 9) were high (highlighted in yellow) what imply they are different species.

|  | sp1 | sp2 | sp3 | sp4 | sp5 | sp6 | sp7 | sp8 | sp9 | *V. esq* |
| --- | --- | --- | --- | --- | --- | --- | --- | --- | --- | --- |
| sp1 | 0.010 |  |  |  |  |  |  |  |  |  |
| sp2 | 0.133 | 0.016 |  |  |  |  |  |  |  |  |
| sp3 | 0.105 | 0.121 | 0 |  |  |  |  |  |  |  |
| sp4 | 0.137 | 0.105 | 0.118 | 0 |  |  |  |  |  |  |
| sp5 | 0.269 | 0.269 | 0.246 | 0.270 | 0 |  |  |  |  |  |
| sp6 | 0.138 | 0.107 | 0.125 | 0.073 | 0.282 | 0.014 |  |  |  |  |
| sp7 | 0.145 | 0.120 | 0.122 | 0.066 | 0.273 | 0.056 | 0.014 |  |  |  |
| sp8 | 0.119 | 0.125 | 0.130 | 0.130 | 0.239 | 0.132 | 0.117 | 0 |  |  |
| sp9 | 0.127 | 0.071 | 0.104 | 0.104 | 0.264 | 0.110 | 0.098 | 0.107 | 0 |  |
| *V.esq* | 0.154 | 0.163 | 0.150 | 0.172 | 0.293 | 0.166 | 0.153 | 0.153 | 0.150 | 0.006 |

Table S3. Pollinating wasp ITS2 nuclear gene sequence differences (Kimura-2-parameter) within (diagonal) and between species (below diagonal). Sequence differences of between *V.esquirolianae* (*V. esq*) and the other *V. javana* pollinating wasps (sp1, 2, 3, 4, 5, 6, 7, 8, 9) were high (highlighted in yellow) what imply they are different species.

|  | sp1 | sp2 | sp3 | sp4 | sp5 | sp6 | sp7 | sp8 | sp9 | *V. esq* |
| --- | --- | --- | --- | --- | --- | --- | --- | --- | --- | --- |
| sp1 | 0 |  |  |  |  |  |  |  |  |  |
| sp2 | 0.103 | 0.004 |  |  |  |  |  |  |  |  |
| sp3 | 0.092 | 0.06 | 0 |  |  |  |  |  |  |  |
| sp4 | 0.104 | 0.034 | 0.060 | 0 |  |  |  |  |  |  |
| sp5 | 0.357 | 0.382 | 0.390 | 0.381 | 0 |  |  |  |  |  |
| sp6 | 0.108 | 0.043 | 0.067 | 0.028 | 0.397 | 0.001 |  |  |  |  |
| sp7 | 0.103 | 0.038 | 0.062 | 0.023 | 0.395 | 0.005 | 0 |  |  |  |
| sp8 | 0.080 | 0.078 | 0.069 | 0.078 | 0.410 | 0.082 | 0.078 | 0 |  |  |
| sp9 | 0.096 | 0.012 | 0.060 | 0.036 | 0.395 | 0.045 | 0.040 | 0.076 | 0 |  |
| *V. esq* | 0.076 | 0.076 | 0.076 | 0.078 | 0.384 | 0.087 | 0.083 | 0.062 | 0.073 | 0 |
